# Supplementary material for: Finding the gap: neuromorphic motion-vision in dense environments
Source: Nat Commun. 2024 Jan 27;15:817. doi: 10.1038/s41467-024-45063-y (PMC10821932; doi:10.1038/s41467-024-45063-y)
Supplement: Supplementary file 1 — Supplementary Information [file 41467_2024_45063_MOESM1_ESM.pdf]

Finding the Gap: Neuromorphic Motion-Vision in Dense Environments

Supplementary Notes 1: sEMD Characterization Setup

To ensure repeatability and reproducibility we recorded the grating in a controlled environment, see Supplementary Figure 1. The Dynamic Vision Sensor (DVS) is mounted in a light sealed box, with a variable distance to the screen. An LED-ring (with 32 LEDs) homogeneously illuminates the DVS's field of view. The LEDs themselves are controlled by an external power-source. The moving screen consists of a thick paper tube, glued together at the ends with double-sided adhesive tape. This tube is clamped over two horizontally mounted cylinders. The lower cylinder is mounted with a floating bearing in the y-direction. The upper cylinder is driven by a stepper motor controlled by an Arduino Uno and translates its movement to the screen. The possible velocities of the screen range from $23\,\mathrm{mm\,s^{-1}}$ to $210\,\mathrm{mm\,s^{-1}}$. The grating itself is printed on dull thick paper forming the paper-tube and stored in the dark to avoid fading out.

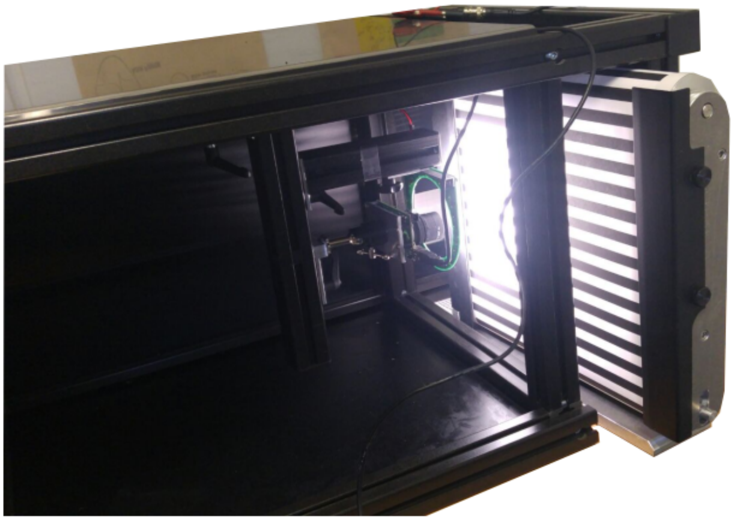

Supplementary Figure 1: Controlled environment for the recordings of the grating. The screen can move either from bottom to top or top to bottom. The upper roll of the screen contraption is driven by an Arduino controlled stepper-motor. The LED - ring illuminates the screen and the event driven camera is located in its center.

Supplementary Notes 2: spiking Elementary Motion Detector (sEMD) Implementation on SpiNNaker

To demonstrate the sEMD's wide range of operation and applicability on multiple platforms, we characterised the model's behaviour on SpiNNaker. We further investigated the sEMD's robustness regarding contrast and illumination. Supplementary Figure 2 a) shows that the model operates well in a wide range of illuminations at 100 % contrast and produces similar velocity tuning curves on SpiNNaker and NEST (see Figure 2e for comparison). Regarding the contrast sensitivity, we found that with the given parameter set, the model reaches half normalized activity at a relative contrast of 45.9 % (see Supplementary Figure 2 b) at 5000 lux illumination and temporal frequency of 5 Hz. Thus the applicability of the model is limited by the occurring contrast but the offered range is still high and can possibly be improved by the implementation of contrast normalisation.

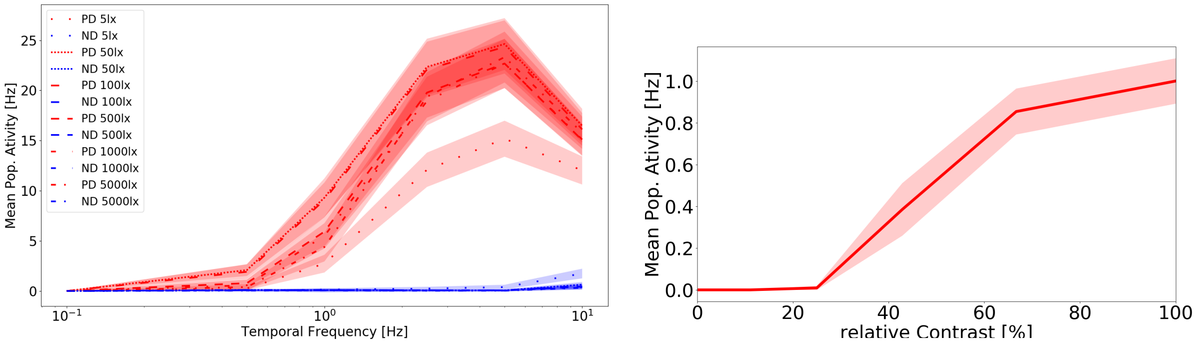

(a) (b)

Supplementary Figure 2: sEMD population response on SpiNNaker for varying illuminations and contrasts. a) Normalised sEMD population preferred direction and null direction response for 100 % contrast and all illuminations from 5 lux to 5000 lux. b) Normalised preferred direction response for 5000 lux illumination over contrasts varying from 0 % to 100 % at a temporal frequency of 5 Hz. For further information on the model parameters see Table 4.

Supplementary Notes 3: The Motion-Vision Network

One very important parameter for obstacle avoidance is the knowledge of the own body size. Orchid bees with a wingspan of approximately 20 mm avoid to pass circular apertures smaller than 25 mm because of a too high collision risk. Some kind of self-representation in the bee's brain has to drive the insect's decision that the gap is too small for it [?]. Similarly, we can tune the connectivity of our Spiking Neural Network (SNN) to indirectly include relevant body size information. Our neural network model needs to consider its own body measures when moving through a gap. This decision process to move or not to move through a gap can be purely driven by the agent's relative perception of the gap. In our obstacle avoidance network this perception is modifiable by a change of the synaptic connections between the integrator neuron population and the inverse WTA population. OF is encoded in a retinotopical map of the integrator neuron population. This neuron population is initially one-to-one connected to the inverse WTA network. By connecting the integrator neuron to its accordant inverse WTA neuron and its closest neighbours the size of the perceived OF caused by an object increases. Therefore, small gaps between objects are closed with increasing number of neighbouring INT to inverse WTA connections which leads to an increase of a perceived gap's minimum size. The angle occupied by a gap has to be bigger than gap_{min} to be considered a movement direction as shown in Equation 1. α_{INT}, the angle of perception of a single INT neuron, amounts to ~2.2° while $n_{connect}$ represents the number of neighbouring connections.

$$gap_{min} = (2 \times n_{connect} + 1) \times \alpha_{sEMD} \quad (1)$$

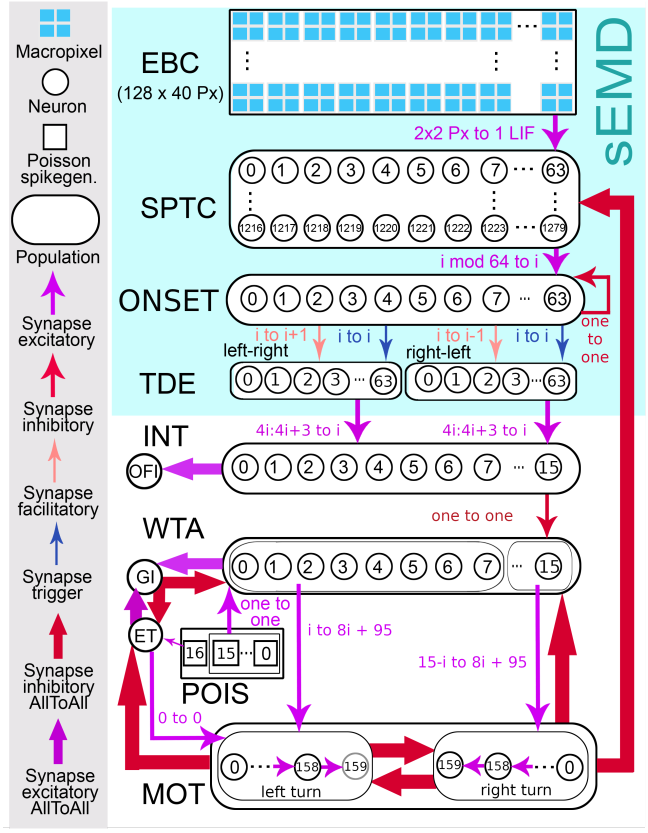

t!

Supplementary Figure 3: Obstacle avoidance network. The macropixels (2x2 pixels) of the Event-Based Camera (EBC) project onto single neurons of the Spatio-Temporal Correlation (SPTC) population removing spatio-temporal uncorrelated events, i.e. noise. Two adjacent SPTC neurons are connected to one Time Difference Encoder (TDE) in the left-right sub-population and the right-left sub-population respectively. Trigger and facilitator connection are opposite in the two populations. The Integrator (INT) population reduces the two dimensional retinotopical map to a one-dimensional map by integrating the spikes of each TDE column onto a single Leaky Integrate and Fire (LIF) neuron. The inverse Winner-Take-All (WTA) population and Escape Turn (ET) population become excited by Poisson spike sources. The WTA mechanism is driven by recurrent suppression through the Global Inhibition (GI) neuron. The two Motor (MOT) populations are activated by a spike in the inverse WTA population. The id of the spiking inverse WTA neuron defines which MOT becomes activated and for how long. 20 different movement directions can be chosen since there are 10 different turning durations for left and right respectively. When the ET neuron spikes the left MOT population becomes activated for the maximal time duration. When the MOT population is inactive the robot moves straight forward collecting apparent motion information. When one MOT population is active the robot turns. All-to-all inhibition between the MOT sub-populations guarantees to disambiguate the steering commands. Inhibition from the MOT to the SPTC population suppresses rotational Optic Flow (OF) input which contains no relative depth information. Inhibition from MOT to inverse WTA hinders the network from taking any new decision during a turn.

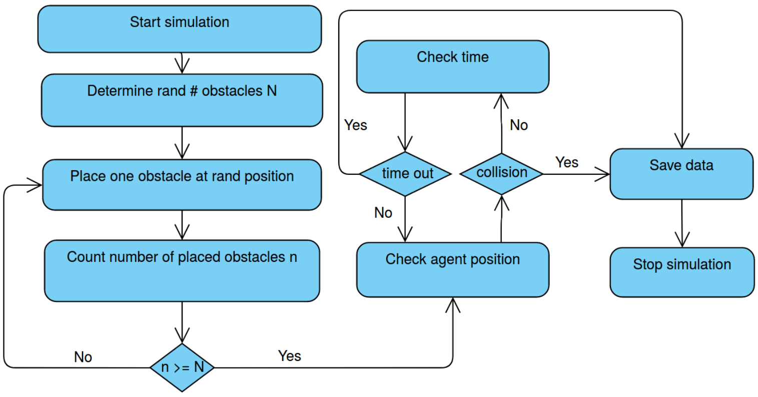

Supplementary Figure 4: State machine to create the cluttered environment and check the agent's obstacle avoidance performance.

Supplementary Notes 4: Gap finding behaviour in cluttered environments

Quantifying the relative motion perception and obstacle avoidance behaviour in controlled environments (see Supplementary Figure 1) allows us to assess the fundamental capabilities of our agent. However, these tests do not fully capture conditions an agent will encounter in the real-world. These conditions include urban areas, indoors as well as outdoor forest environments. A simple, yet effective test environment thus should be characterised with variable amount of clutter, i.e. obstacle density, of vertical obstacles placed in a random configuration. We introduced the agent in an arena and varied the obstacle density from 0% up to 38% and measured the maximum distance (see Supplementary Figure 5) as a function of increasing obstacle density. The minimum distance of the robot from the starting point first increases for fixed and adaptive velocity, and then goes down. The low minimum distance for low obstacle densities is caused by the random movement algorithm of the agent. Without any objects the robot will move ranadomly in all directions, therefore staying close to the start point. With increasing obstacle densities, pathways form in teh environment and the robot starts following these pathways leading to higher distances from teh starting point. When the obstacle density further increases the pathways close and the robot can not leave the starting point anymore which causes a reduction of the maximum distance. For a fixed velocity the success rate goes slightly down for higher obstacle densities while for an adaptive velocity it stays close to 100 percent (see Supplementary Figure 6). Interestingly, due to the employed adaptive movement strategy the agent's velocity decreases almost linearly with increasing obstacle density (see Supplementary Figure 7). This adaptive behaviour ensures that despite high clutter the agent successfully identifies gaps in the environment and steers towards them and consequently avoids collisions with its surrounding.
The agent's movement behaviour shows the intended pattern, a division into saccadic turns and intersaccadic straight movements (see Supplementary Figure 8) .

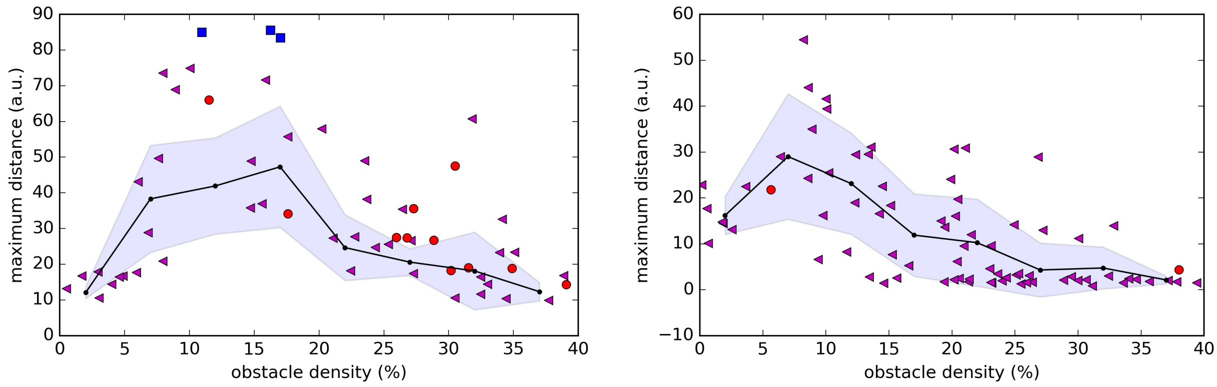

(a) (b)

Supplementary Figure 5: Agent's maximum distance to the start location with fixed (a) and adaptive (b) velocity for the robot data from Figure 4e.

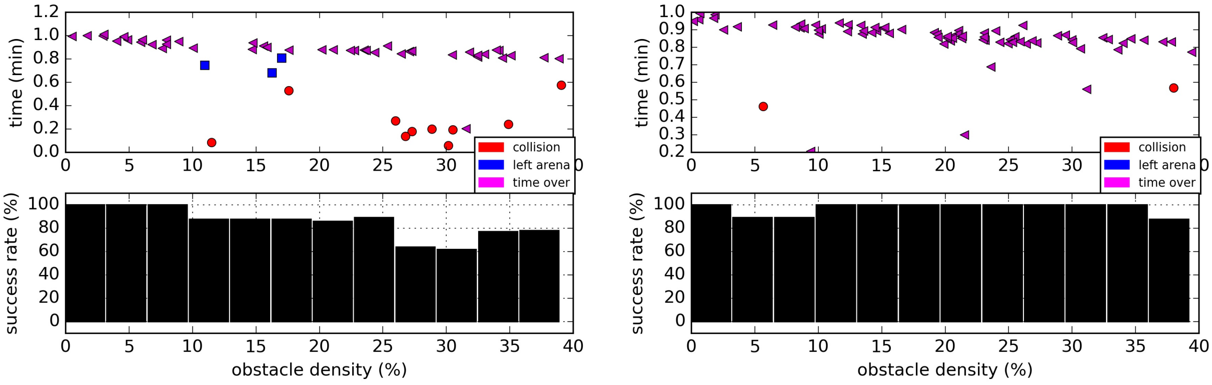

(a) (b)

Supplementary Figure 6: Agent's behaviour in cluttered environments with the parameters from Table 5 and 6 moving with a (a) fixed intersaccadic velocity and (b) adaptive intersaccadic velocity. Top: Simulation time at which the simulated robot leaves the arena, collides or the simulation is over. Bottom: Agent's success rate, hence number of runs without collisions.

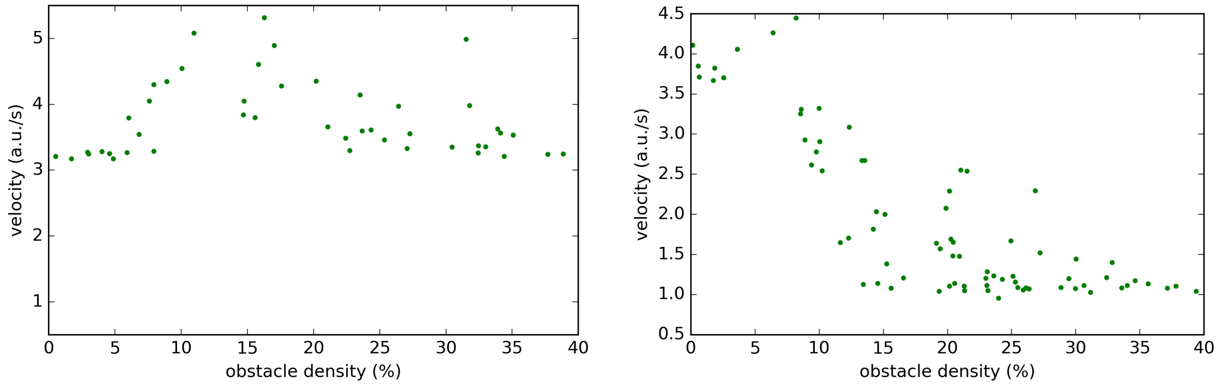

(a) (b)

Supplementary Figure 7: Agent's mean velocity over obstacle density with (a) fixed and (b) adaptive velocity. Variations in the mean velocity in (a) are caused by variations in the number and duration of saccadic and intersaccadic movements.

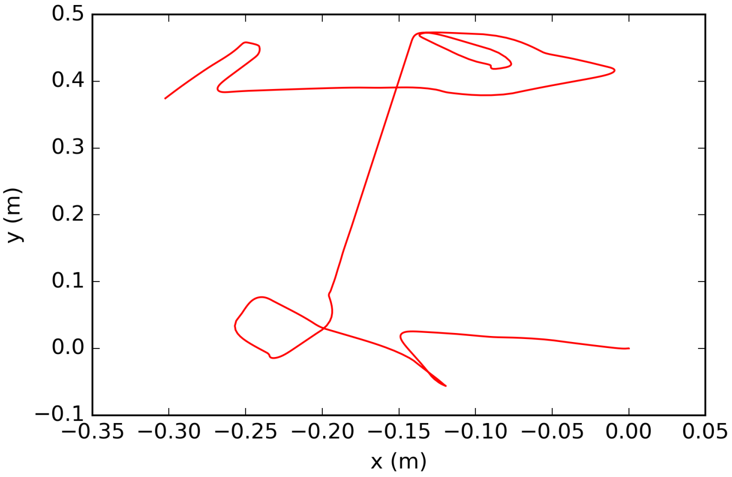

Supplementary Figure 8: Agent's movement trajectory for two seconds in an environment with low clutter, similar run than Figure 4a.

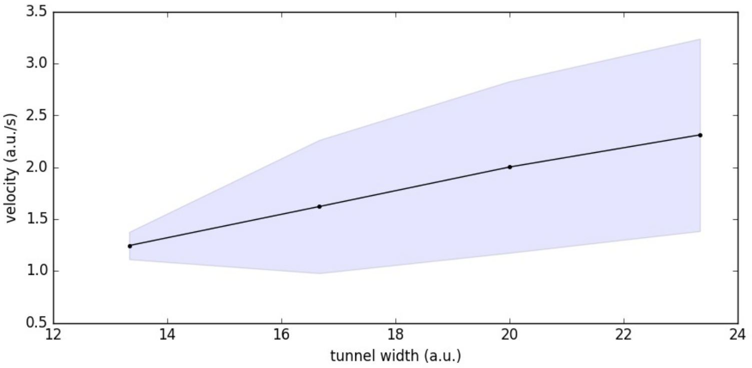

Supplementary Figure 9: Robot velocity in the simulated tunnel for different tunnel width.

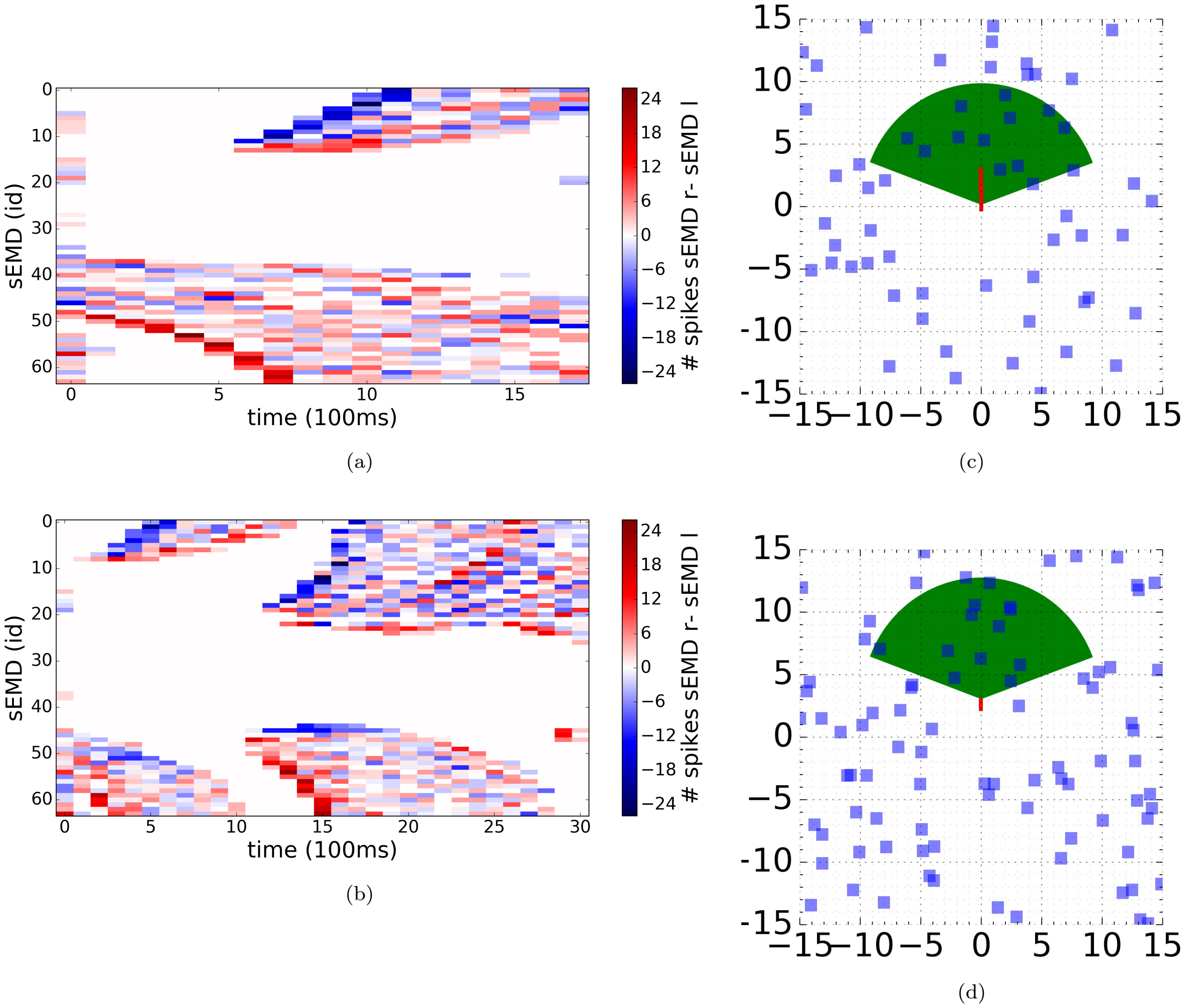

Supplementary Figure 10: sEMD activity over time while the agent is moving straight through a cluttered environment. a-b) difference in left and right sEMD activity while the agent is moving straight through two different cluttered environments. The direction of OF is correctly detected at the edges of the objects. However, a spatial frequency of the objects higher than the temporal frequency of the OF makes the detection of the direction of OF inside the object imprecise. This is not a problem for the algorithm used in this paper since only the strength of OF is relevant for the obstacle avoidance task. c-d) Cluttered environment trough which agent is moving. Subplot a) and c) as well as b) and d) show data from the same run respectively.

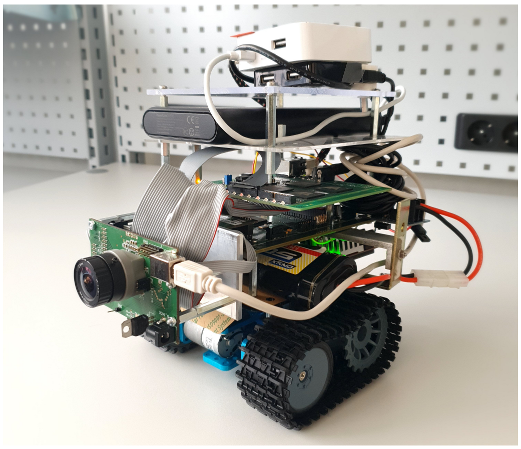

(a)

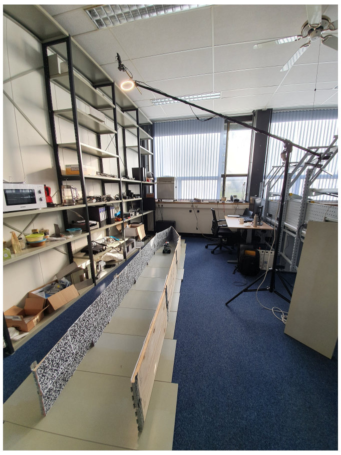

(b)

Supplementary Figure 11: Robot and setup to conduct the real world experiment. a) The robot received visual input from the Dynamic Vision Sensor. The event-based camera sends its events to a SpiNN-3 board which simulates a version of the obstacle avoidance network. b) Experimental setup for the corridor centering experiment. The corridor walls were covered with random checkerboard patterns. The ground of the arena consisted of metal plates since the motors of the robot are not strong enough to move the vehicle on the carpet. A webcam and a light-ring mounted on a tripod above the arena were used to film the robot and illuminate the arena.

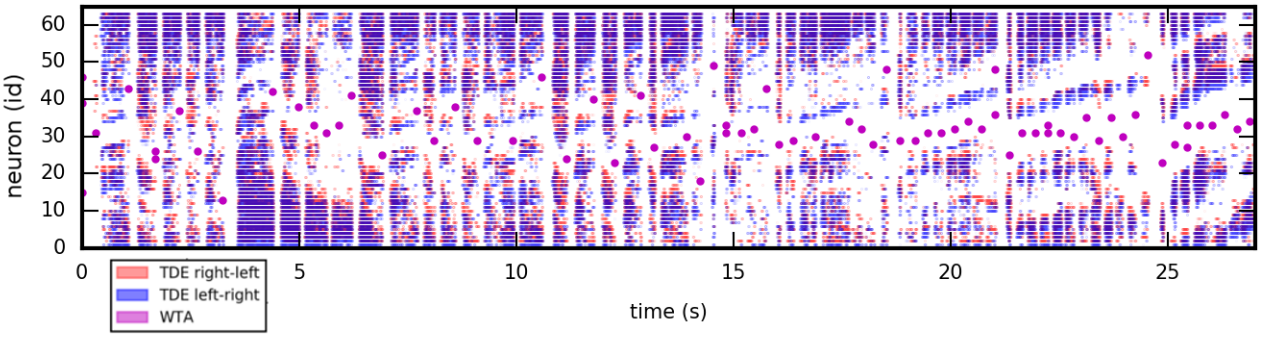

(a)

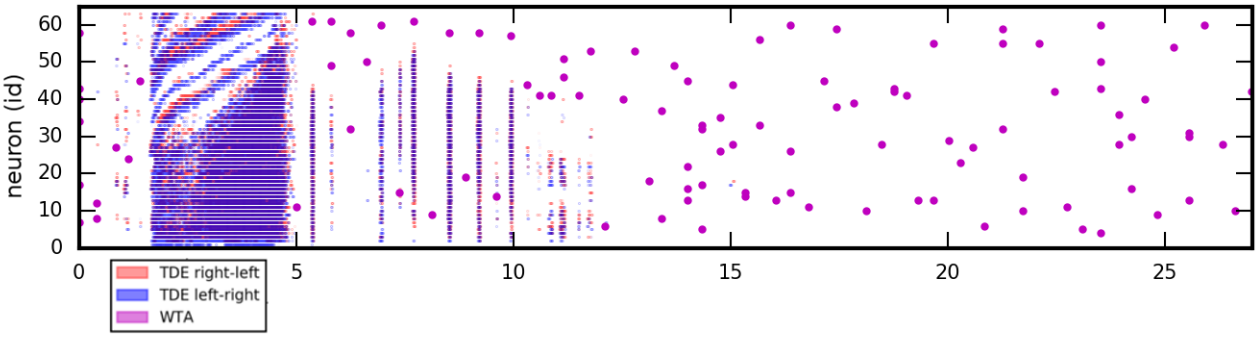

(b)

Supplementary Figure 12: Robot spiking activity examples for the real world corridor centering experiment shown in Supplementary Figure 11. (a) Successful run in wide corridor. (b) Crash in wide corridor. The robot does not find an obstacle free direction indicated by a spike in the WTA population after the first initial turn. Hence, it crashes.

1 Tables

printed Contrast	Temporal Frequency (Hz)	Illumination (lux)
0	0.1	5
0.2	0.5	50
0.4	1.0	100
0.6	2.5	500
0.8	5.0	1000
1.0	10.0	5000

Supplementary Table 1: Parameters of grating recordings. Three four second recordings were made for each possible parameter-combination.

Simulation	Figures	Repetitions	Real time duration (min)
Clutter adaptive velocity	2f,i, A.5, A.7a	100	360
Clutter fixed velocity	2i, A.6	70	360
Corridors	2g,j,k,l	10 per corridor width	60
Real World Corridor	2d, A.9.	10 per corridor width	-
Gaps	2e,h	10 per gap size	180

Supplementary Table 2: Parameters of simulations and real world experiment.

Name	Type	C_m (nF)	tau_m (ms)	tau_{ref} (ms)	v_{reset} (mV)	v_{rest} (mV)	v_{thresh} (mV)	tau_{syn_E} (ms)	tau_{syn_I} (ms)	I_{offset} (nA)	Popsize ($col \times row$)	#Pop
DVS	SSA										128×128	1
SPTC	LIF	0.25	20	1	-85	-60	-50	20	20	0	32×32	1
sEMD	TDE	0.25	20	1	-85	-60	-50	20	20	0	32×32	2

From	To	Weight (nA)	Connection type	Synapse type	delay (ms)
DVS	SPTC	0.2	(int(i/(128*4)*32) +int(i % (128*4) / 3) to i)	excitatory	1
SPTC	TDE top-bottom	0.2	one_to_one	facilitator	1
SPTC	TDE top-bottom	0.2	i to i+32	trigger	1
SPTC	TDE bottom-top	0.2	one_to_one	trigger	1
SPTC	TDE bottom-top	0.2	i+32 to i	facilitator	0.1

Supplementary Table 3: Neuron Parameters and Connections on SpiNNaker for sEMD characterization.

Name	Type	C_m (nF)	tau_m (ms)	tau_{ref} (ms)	v_{reset} (mV)	v_{rest} (mV)	v_{thresh} (mV)	tau_{syn_E} (ms)	tau_{syn_I} (ms)	I_{offset} (nA)	Popsize ($col \times row$)	#Pop
DVS	SSA										128×128	1
SPTC	LIF	0.25	35	1	-70	-65	-40	30	1	0	64×64	1
sEMD	TDE	0.25	30	1	-70	-65	-40	100	50	0	64×20	2
INT	LIF	0.25	20	1	-70	-65	-40	5	5	0	64×1	2
WTA	LIF	0.25	30	1	-70	-65	-40	100	50	0	64×1	1
GI	LIF	0.25	30	2	-68	-65	-50	40	5	0	1×1	1
MOT	LIF	0.25	20	2	-68	-65	-50	5	5	0	96×1	2
OUTPUT	LIF	0.25	20	2	-68	-65	-50	5	5	0	512×1	1
Name	Type					Rate	(Hz)				Popsize	$\#Pop$
POIS1	Spike Source					50					64×1	1

Supplementary Table 4: Neuron Parameters on SpiNNaker for real-world corridor centering experiment.

Name	Type	E_L (mV)	C_m (pF)	tau_m (ms)	t_{ref} (ms)	tau_{syn_exc} (ms)	tau_{syn_inh} (ms)	V_{th} (mV)	V_{reset} (mV)	V_m (mV)	Popsize ($col \times row$)	$\#Pop$
SPTC	LIF	-60.5	25	20	1	10	10	-60	-60.5	-60.5	64×20	1
ONSET	LIF	-70.0	250	150	1	10	10	-60	-70	-70	64×1	2
sEMD[0-31]	TDE	-60.0	250	10	1	10	23-i/2	-30	-85	-60	32×1	2
sEMD[32-63]	TDE	-60.0	250	10	1	10	$8 + i/2$	-30	-85	-60	32×1	2
INT	LIF	-70	250	30	1	20	10	-60	-70	-70	16×1	2
WTA	LIF	-65	250	20	1	5	80	-50	-68	-65	16×1	1
MOT	LIF	-65	250	20	2	5	5	-50	-68	-65	160×1	2
GI	LIF	-65	250	30	2	40	5	-50	-68	-65	1×1	1
OFI	LIF	-80	250	200	1	100	30	-40	-80	-75	1×1	1
ET	LIF	-65	250	20	1	5	80	-50	-68	-65	1×1	1
Name	Type		Rate	(Hz)							Popsize	$\#Pop$
POIS1	Spike Source		100								16×1	1
POIS2	Spike Source		100								1×1	1

Supplementary Table 5: Neuron Parameters from Neurorobotics Platform NEST network.

From	To	Weight (nA)	Connection type	Synapse type	delay (ms)
DVS	SPTC	0.5	(i and i+1 and i+128 and i+ 129) to i	excitatory	1
SPTC	TDE left-right	2	34*64+i+1 to i	trigger	1
SPTC	TDE left-right	2	34*64+i to i	facilitator	1
SPTC	TDE right-left	2	34*64+i+1 to i	facilitator	1
SPTC	TDE right-left	2	34*64+i to i	trigger	1
TDE right-left	INT right-left	3	i mod 64 to i	excitatory	1
TDE left-right	INT left-right	3	i mod 64 to i	excitatory	1
INT right-left	WTA	1	one_to_one	inhibitory	1
INT right-left	WTA	0.75	i to $i\pm$ 1	inhibitory	1
INT right-left	WTA	0.5	i to $i\pm$ 2	inhibitory	1
INT right-left	WTA	0.3	i to $i\pm$ 3	inhibitory	1
INT right-left	WTA	0.25	i to $i\pm$ 4	inhibitory	1
INT left-right	WTA	1	one_to_one	inhibitory	1
INT left-right	WTA	0.75	i to $i\pm$ 1	inhibitory	1
INT left-right	WTA	0.5	i to $i\pm$ 2	inhibitory	1
INT left-right	WTA	0.3	i to $i\pm$ 3	inhibitory	1
INT left-right	WTA	0.25	i to $i\pm$ 4	inhibitory	1
WTA(0-5)	MOT1	10	i to 38	excitatory	1
WTA(6-31)	MOT1	10	i to 2i + 32	excitatory	1
WTA(32-57)	MOT2	10	63 - i to 2i + 32	excitatory	1
WTA(58-63)	MOT2	10	i to 38	excitatory	1
WTA	GI	15	all_to_all	excitatory	1
GI	WTA	15	all_to_all	inhibitory	1
MOT1	WTA	5	all_to_all	inhibitory	1
MOT1	MOT2	10	all_to_all	inhibitory	1
MOT1	SPTC	50	all_to_all	inhibitory	1
MOT1	MOT1	2	i to i + 1	excitatory	4
MOT1	MOT1	10	one_to_one	inhibitory	1
MOT1	OUTPUT	10	4i to 188	excitatory	1
MOT1	OUTPUT	10	4i to 314	excitatory	1
MOT1	OUTPUT	10	95 to 336	excitatory	1
MOT1	OUTPUT	10	95 to 65	excitatory	1
MOT2	WTA	5	all_to_all	inhibitory	1
MOT2	MOT1	10	all_to_all	inhibitory	1
MOT2	SPTC	50	all_to_all	inhibitory	1
MOT2	MOT2	2	i to i + 1	excitatory	4
MOT2	MOT2	10	one_to_one	inhibitory	1
MOT2	OUTPUT	10	4i to 60	excitatory	1
MOT2	OUTPUT	10	4i to 442	excitatory	1
MOT2	OUTPUT	10	95 to 336	excitatory	1
MOT2	OUTPUT	10	95 to 65	excitatory	1
POIS1	WTA	2	one_to_one	excitatory	1

Supplementary Table 6: Neuron connections from SpiNNaker for real-world corridor experiment. Note: There might be slight differences in the connection scheme when comparing Figure 3 with this table. This is because Figure 3 only serves for demonstration purposes. Always use the connections from this table to rebuild the network for the robot.

From	To	Weight (nA)	Connection type	Synapse type	delay (ms)
DVS NRP	SPTC	default	(i and i+1 and i+128 and i+ 129) to i	excitatory	0.1
DVS real world	SPTC	0.002	(i and i+1 and i+128 and i+ 129) to i	excitatory	0.1
SPTC	ONSET	0.2	i mod 64 to i	excitatory	0.1
ONSET	ONSET	-2	one_to_one	inhibitory	0.1
ONSET	TDE left-right	4	one_to_one	trigger	0.1
ONSET	TDE left-right	4	i to i+1	facilitator	0.1
ONSET	TDE right-left	4	one_to_one	facilitator	0.1
ONSET	TDE right-left	4	i+1 to i	trigger	0.1
TDE right-left	INT	0.5	4i:4i+3 to i	excitatory	0.1
TDE left-right	INT	0.5	4i:4i+3 to i	excitatory	0.1
INT	WTA	-0.1	one_to_one	inhibitory	0.1
INT	OFI	10	all_to_all	excitatory	0.1
WTA(0-7)	MOT1	10	i to 8i + 95	excitatory	0.1
WTA(8-15)	MOT2	10	15-i to 8i + 95	excitatory	0.1
WTA	GI	10	all_to_all	excitatory	0.1
ET	MOT1	10	0 to 0	excitatory	0.1
ET	GI	10	all_to_all	excitatory	0.1
GI	ET	-10	all_to_all	inhibitory	0.1
GI	WTA	-10	all_to_all	inhibitory	0.1
MOT1	WTA	-30	all_to_all	inhibitory	0.1
MOT1	ET	-30	all_to_all	inhibitory	0.1
MOT1	MOT2	-10	all_to_all	inhibitory	0.1
MOT1	Sensors	-30	all_to_all	inhibitory	0.1
MOT1	MOT1	10	i to i + 1	excitatory	10
MOT1	MOT1	-10	one_to_one	inhibitory	0.1
MOT2	WTA	-30	all_to_all	inhibitory	0.1
MOT2	ET	-30	all_to_all	inhibitory	0.1
MOT2	MOT1	-10	all_to_all	inhibitory	0.1
MOT2	Sensors	-30	all_to_all	inhibitory	0.1
MOT2	MOT2	10	i to i + 1	excitatory	10
MOT2	MOT2	-10	one_to_one	inhibitory	0.1
POIS1	WTA	1	one_to_one	excitatory	0.1
POIS2	ET	0.3	one_to_one	excitatory	0.1

Supplementary Table 7: Neuron connections from NEST network used in the neurorobotics platform. Note: There might be slight differences in the connection scheme when comparing Figure 3 with this table. This is because Figure 3 only serves for demonstration purposes. Always use the connections from this table to rebuild the network.
