# Supplementary material for: Finding the gap: neuromorphic motion-vision in dense environments
Source: Nat Commun. 2024 Jan 27;15:817. doi: 10.1038/s41467-024-45063-y (PMC10821932; doi:10.1038/s41467-024-45063-y)
Supplement: Supplementary file 3 — Description of Additional Supplementary Files [file 41467_2024_45063_MOESM3_ESM.pdf]

**Description of Additional Supplementary Files**

**File Name:** Supplementary Movie 1
**Description:** Finding the Gap: We introduce the motion-vision algorithm. After that we show examples of the real-world corridor experiments and the robot in cluttered environments.
